# Superior Mesenteric Artery Syndrome Following Tubercular Intestinal Perforation

**DOI:** 10.7759/cureus.4506

**Published:** 2019-04-20

**Authors:** Hitesh Gupta, Ankit Agrawal, Akshant A Pathak

**Affiliations:** 1 General Surgery, Guru Teg Bahadur Hospital/University College of Medical Sciences, New Delhi, IND; 2 Internal Medicine, Saint Peter's University Hospital - Rutgers Robert Wood Johnson Medical School, New Brunswick, USA

**Keywords:** superior mesenteric artery syndrome, tuberculosis, small intestine perforation

## Abstract

Superior mesenteric artery (SMA) syndrome is a rare clinical entity characterized by the compression of third part of the duodenum between the superior mesenteric artery and abdominal aorta due to loss of intervening mesenteric fat pad. This article reports a case of a patient operated for tubercular intestinal perforation following which she developed postprandial abdominal pain and recurrent vomiting in the postoperative period. Contrast enhanced computed tomography (CECT) of abdomen was done which showed gastric dilatation extending till the third part of duodenum with decreased aorto-mesenteric angle, compatible with the diagnosis of SMA syndrome. The patient was managed conservatively on total parenteral nutrition. Six weeks after the surgery, patient’s symptoms resolved completely on conservative management and recovered without any need of surgical intervention. This case illustrates the pathogenesis of SMA syndrome in the setting of severe weight loss caused by tuberculosis superimposed by the catabolic state of surgery leading to rapid loss of mesenteric fat which was successfully managed conservatively.

## Introduction

Superior mesenteric artery (SMA) syndrome is a rare gastrointestinal disorder caused by the compression of third part of the duodenum between superior mesenteric artery and abdominal aorta. It was first described by Rokitansky [1] and has a reported incidence of 0.013 to 0.3% in general population [2]. Duodenal compression occurs due to decrease in the angle and distance between an abdominal aorta and superior mesenteric artery resulting in obstruction. This article reports a case of SMA syndrome due to severe weight loss following surgery of tubercular intestinal perforation.

## Case presentation

**Figure 1:**
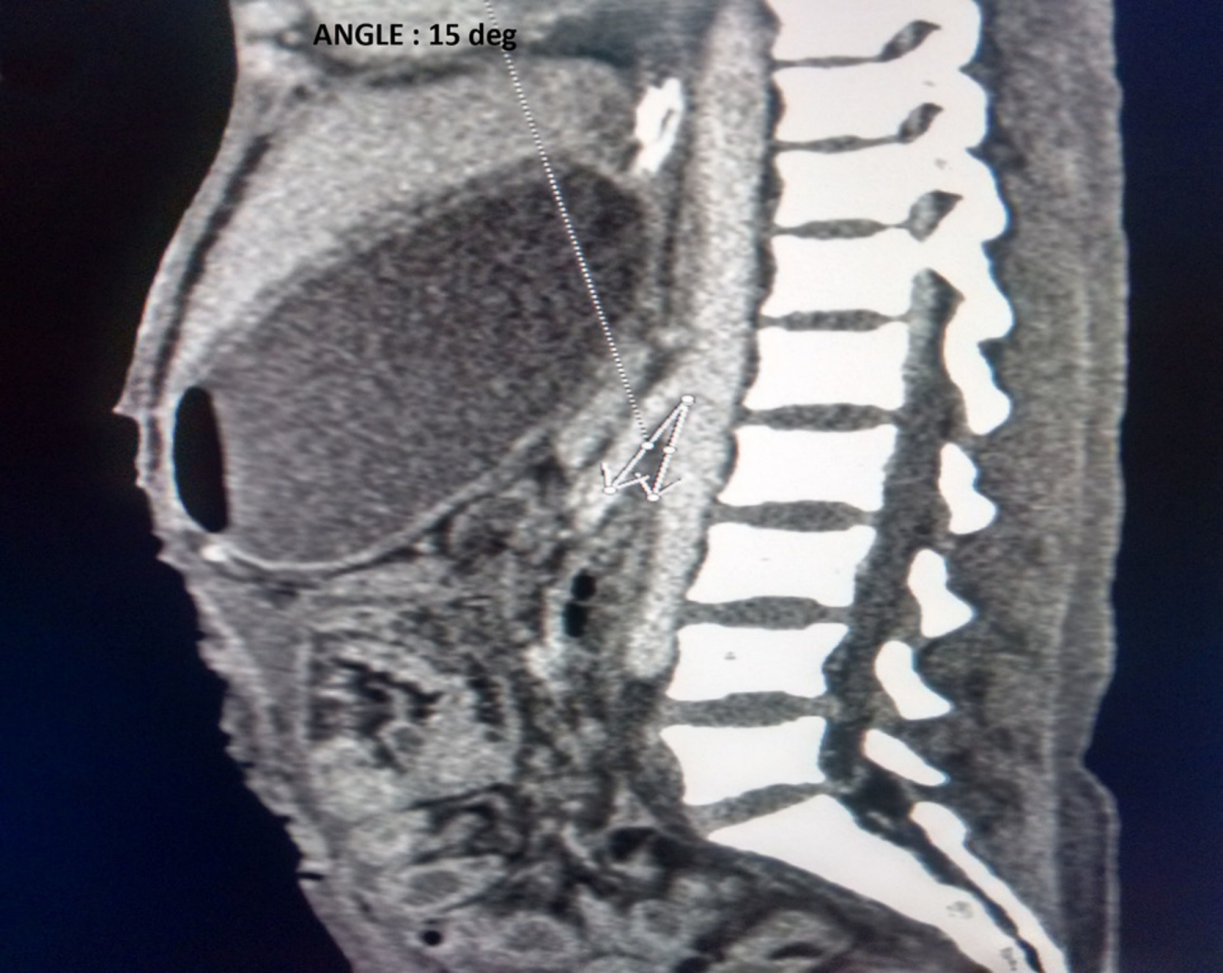
Sagittal section of contrast enhanced computed tomography of abdomen showing decreased aortomesenteric angle of 15 degrees (white arrows).

A 20-year-old woman presented to the emergency department with complaints of severe abdominal pain, multiple episodes of vomiting and obstipation for one day. She was a known case of abdominal tuberculosis and had received anti-tubercular treatment for two months. Vital signs showed a blood pressure of 100/64 mm Hg, heart rate of 110 per minute, respiratory rate of 18 per minute, oxygen saturation of 96% on room air and a temperature of 100.5°F. She was cachectic with a weight of 28 kg which was less than 5th percentile for gender and age matched normal population. Physical examination revealed diffuse abdominal tenderness and rigidity. Her erect abdominal radiography showed gas under right dome of the diaphragm. Clinical diagnosis of perforation peritonitis was made and the patient was wheeled to the operating room for emergent laparotomy. Intraoperatively, a 3 cm x 2 cm single ileal perforation was found 1 foot proximal to the ileocecal junction, with unhealthy bowel margins and multiple mesenteric lymphadenopathy. Resection of perforated bowel segment with proximal ileostomy and mucus fistula of distal ileum was performed. Later, histopathology report confirmed tubercular etiology showing caseous necrosis in mesenteric lymph nodes and epithelioid granuloma in ulcer edge biopsy. Two days after the surgery, the patient was started on oral feeds and was accepting meals orally subsequently. Ten days following surgery, the patient had multiple episodes of bilious vomiting with stoma output decreasing to less than 100 mL per day. Her abdomen remained flat with no increase in bowel sounds. Abdominal radiography showed dilated stomach with no air fluid levels. Oral feeds were stopped and a nasogastric tube was placed for drainage of gastric contents. A contrast enhanced computed tomography (CECT) abdomen was done to determine the cause of obstruction which revealed a decreased aortomesenteric angle of 15° (Figure 1).

**Figure 2:**
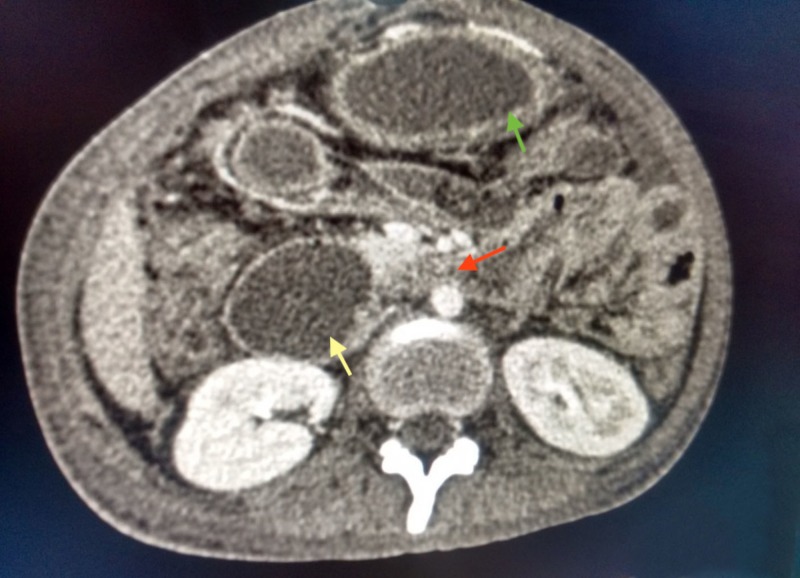
Axial view of contrast enhanced computed tomography of abdomen showing compression of third part of the duodenum (red arrow) with gastric dilation (green arrow) and proximal duodenal dilation (yellow arrow).

As a result of this decreased angle, an abrupt collapse of the third part of the duodenum was seen along with proximal duodenal and stomach dilation (Figure 2).

**Figure 3:**
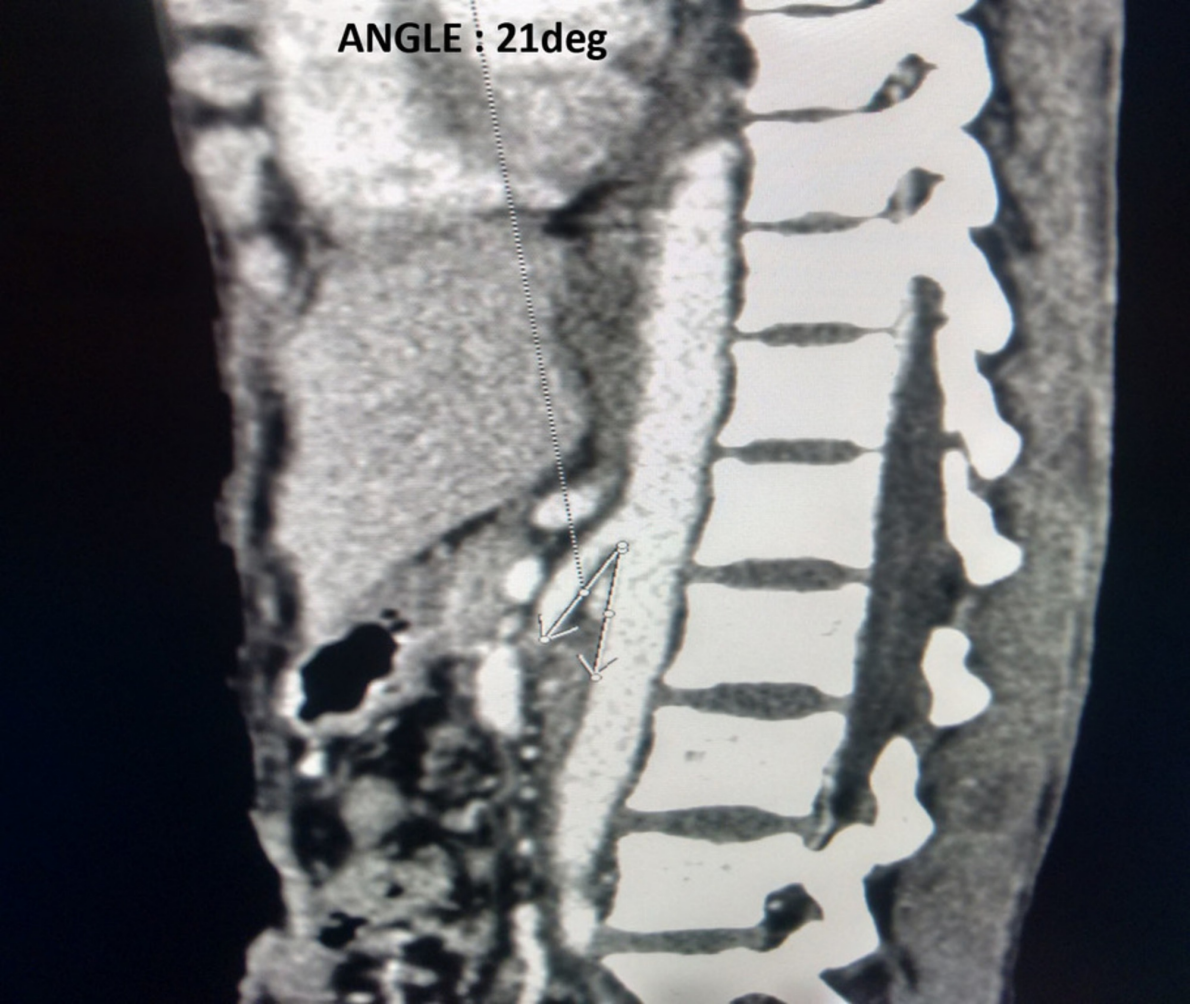
Sagittal section of contrast enhanced computed tomography of abdomen showing increased aortomesenteric angle of 21 degrees (white arrows) after weight gain.

Based on the clinical features and CECT findings, diagnosis of SMA syndrome was made and the patient was managed conservatively. The patient was not tolerating enteral feeds and no relief of postprandial abdominal pain was noticed even on changing to left lateral decubitus or prone position. Considering the patient’s nutritional condition, total parenteral nutrition (TPN) was started. Anti-tubercular drugs were continued with oral sips. She was started on 1500 kcal/day (50 kcal/kg/day), taking care of refeeding syndrome and was gradually titrated up to provide maximal caloric support. Monitoring of weight, blood glucose, electrolytes, liver function test, and lipid profile was done to prevent any TPN complications. A good clinical response was seen after starting TPN. The patient’s weight gradually increased to 34 kg in three weeks and gradually the stoma output increased and nasogastric drainage decreased. TPN was tapered off gradually with concurrent starting of oral feeds. On discharge, the patient was advised a high caloric and protein diet with small frequent meals. A follow-up CECT was done after six weeks from discharge which showed an increase in aortomesenteric angle from 15° to 21° (Figure 3) with the patient being asymptomatic and a total weight gain of 12 kg post-surgery.

## Discussion

SMA syndrome, also known as Wilkie’s syndrome, is a rare clinical entity. It is most commonly seen in young females between the age group of 10-40 years, characterized by the compression of third part of the duodenum due to decrease in the angle between the abdominal aorta and the overlying superior mesentery artery. Normal aortomesenteric angle of 38°-56° and aortomesenteric distance of 10-28 mm is reduced to 6°-16° and 2-8 mm in SMA syndrome, respectively [2]. The commonly described causes of this syndrome are severe weight loss due to anorexia nervosa, malabsorption syndrome, diabetes mellitus, catabolic states like malignancy, burns and after application of spica cast. SMA syndrome generally presents with repeated episodes of vomiting, nausea, postprandial epigastric pain which is relieved after changing to left lateral or prone position. The suspicion of SMA syndrome rests on clinical symptoms and is diagnosed based on a combination of clinical picture and imaging studies. CECT abdomen can show duodenal obstruction with reduced aortomesenteric angle and aortomesenteric distance. It can also assess the retroperitoneal fat content and rule out the other causes of obstruction. Treatment of SMA syndrome begins with immediate decompression of stomach through nasogastric drainage and correction of electrolyte disorder. After stabilization, small frequent meals are started with change of position to left lateral decubitus after eating. A nasojejunal feeding tube can be passed distal to site of obstruction to provide enteral feeds. In severe cases in which enteral feeding is not tolerated, TPN may be required. The main goal of treatment in SMA syndrome is weight gain which causes restoration of the duodenal fat pad. Surgery is required in cases not responding to conservative treatment. It consists of open or laparoscopic duodenojejunostomy, gastrojejunostomy or Strong’s procedure. Duodenojejunostomy is considered as a procedure of choice [3].

To the best of our knowledge, there were only two cases of SMA syndrome caused by tuberculosis reported in the past [4,5]. In none of the cases reported has SMA syndrome occurred after a surgery. Ours is seemingly the first case of SMA syndrome described after surgery for tubercular intestinal perforation and managed conservatively. The falling stoma output and later return to normal levels provided an important and unique marker in tracking the disease progression. The reason of SMA syndrome in our case is due to severe weight loss caused by tuberculosis superimposed by the catabolic state of surgery leading to loss of retroperitoneal fat. Tuberculosis can itself cause duodenal obstruction due to extrinsic compression by adjacent lymph nodes or duodenal stricture mimicking SMA syndrome [6]. However, this was absent in our scenario.

## Conclusions

As seen in our case, full restoration to normal anatomical values of aortomesenteric angle and distance was not needed for restoration of normal duodenal function. Indeed, even as normal function returned, the radiological findings were still in the described range of SMA syndrome. Based on this, a conclusion can be drawn that SMA syndrome may be present radiologically in many patients, however overt clinical symptoms appear in a select few with the exact idiosyncratic factors being still unclear.
